# Comparative analysis of maze complexity: implications for adult hippocampal neurogenesis

**DOI:** 10.3389/fnins.2026.1766823

**Published:** 2026-03-09

**Authors:** Mohamed Hesham Khalil

**Affiliations:** Department of Architecture, University of Cambridge, Cambridge, United Kingdom

**Keywords:** adult hippocampal neurogenesis, environmental enrichment, hippocampus, human, maze, mice, rat, spatial complexity

## Abstract

Adult hippocampal neurogenesis persists throughout the lifespan in mammals and can be enhanced by environmental enrichment, including spatial complexity. Whilst maze-based enrichment has been suggested to increase neurogenesis in rodents, the relative complexity of different maze architectures has not been quantified, limiting translational research to human environments. This study used the Architectural Spatial Complexity Index (A-SCI), a novel tool, to compare spatial complexity across 16 rodent maze configurations, including mazes suggested to increase neurogenesis. Results confirm that the A-SCI significantly differentiates environmental enrichment from standard housing, whilst post-hoc analysis suggests no significant difference between maze-based and object-based enrichment, consistent with previous research reporting almost similar effects on cortical thickness. Comparative analysis revealed substantial variation in complexity across rodent maze architectures. Future research can use the A-SCI tool to validate the effect of maze complexity on adult hippocampal neurogenesis, while the Hampton Court maze may be a promising translational paradigm from rodents to humans.

## 1 Introduction

The effectiveness of mazes on adaptive brain plasticity dates back to before spatial complexity was studied as a form of environmental enrichment for adult hippocampal neurogenesis in mice. Despite that subsequent research using spatial complexity relied on tunnels, toys, running wheels, and other points of interest in an open field, mazes remain the most comparable to building layouts, landscape architecture and city street network for humans whose brains also demonstrate adult hippocampal neurogenesis. Addressing this gap is critical because testing the impact of spatial complexity on hippocampal neurogenesis in humans is either invasive or not feasible, while relying on animal models poses a limitation of irrelevant representation of spatial complexity. Therefore, this article presents the architectural spatial complexity index as a pro-neurogenic environmental enrichment assessment tool for environments of both animals and humans.

Rosenzweig et al. (1978) tested whether brain changes from enriched environments were affected by environmental complexity independently from social enrichment. They housed groups of 12 male rats for 30 days in conditions with constant group size but systematically varying physical environments: (a) GC (Group Condition)—large cage (75 × 75 × 45 cm) with no objects; (b) SM (Simple Maze)—same-sized cage with a plastic maze box whose pattern stayed fixed throughout; (c) CM (Complex Maze)—same-sized cage with a maze whose barrier pattern changed daily for 29 days; (d) EC (Enriched Condition—same-sized cage with about 6 varied stimulus objects from a pool of 25, with objects changed every few days and rats rotated between cages daily; and (e) SNE (Semi-natural Environment)—outdoor 9 × 9 meter concrete pit with ~30 cm of earth, with stones, branches, wood pieces, and growing weeds on the surface. All groups were compared to isolated littermates in small cages (32 × 20 × 20 cm). Brain measures increased progressively with environmental complexity: GC showed the smallest differences from isolation (2.7% increase in total cortex weight), SM showed 4.1% increase in total cortex, CM showed 5.2% increase in total cortex, EC showed 5.5% increase in total cortex, and SNE showed the greatest effects (7.4% increase in total cortex). This demonstrated that physical environmental features, not just social housing, drive structural and chemical brain changes, with the outdoor semi-natural environment producing the greatest effects.

Where both CM and EC show almost similar outcomes in the study by Rosenzweig et al. (1978), the evidence supports the argument in this paper that complex mazes can be equally effective for adaptive plasticity, and hypothetically for adult hippocampal neurogenesis. To support this conceptual hypothesis, a couple of studies support that mazes increase adult hippocampal neurogenesis despite the confounding effect of including running wheels. First, Fares et al. (2013) demonstrated that rats housed in the Marlau™ cage showed increased hippocampal neurogenesis, with a greater number of BrdU-positive cells in the dentate gyrus granule cell layer, the majority of which differentiated into neurons. The Marlau™ cage consisted of a series of mazes, the configuration of which is changed regularly three times per week, with the maze separating food and water compartments, thus incorporating both complexity and novelty into the design. The cage also included running wheels to promote increased voluntary exercise and large surfaces to explore. The authors noted that the change in maze configuration was inspired by the environmental complexity and training paradigm. However, whilst the authors acknowledged that voluntary exercise is one of the behaviors promoted by the Marlau™ cage and discussed how voluntary exercise can increase hippocampal transcript levels of growth factors like IGF-1, they did not experimentally separate the effects of maze exploration from running wheel activity on neurogenesis. The study design did not include conditions that isolated these two components to determine their independent contributions to the observed neurogenic effects. Second, Crouzier et al. (2018) similarly found that mice trained in the Hamlet test showed increased hippocampal neurogenesis, with significant increases in both cell proliferation (measured by BrdU and Ki67 immunolabelling) and neuronal maturation (measured by doublecortin labelling). The Hamlet consisted of a central agora with streets expanding in a star shape towards five functionalized houses, one of which (the Run house) contained a running wheel, whilst the maze configuration itself was not changed during training sessions. The authors stated that hippocampal plasticity linked to new memories or training to a novel environment relies intrinsically on the DG, and concluded that Hamlet training clearly stimulated hippocampal plasticity and neurogenesis. The authors acknowledged that neurogenesis is increased by learning and by enriched environment, but they did not experimentally dissociate the contribution of spatial navigation through the maze from physical exercise via the running wheel. Neither study explicitly attributed the neurogenic effects solely to maze exploration or running wheel activity, nor did either implement experimental controls (such as maze-only or wheel-only conditions) to differentiate between these confounding factors.

Strong claims about an independent effect of maze complexity on neurogenesis can be made, however, for two reasons that build on each other. First, Rosenzweig et al. (1978) reported that mazes and environmental enrichment using objects both resulted in almost similar outcomes. Second, assuming both enrichment mechanisms are equal in effect, Van Praag et al. (1999) explored which component of environmental enrichment drives increased neurogenesis by separating out different factors, and the results suggest that enrichment and running both support adult hippocampal neurogenesis but arguably in different ways. They assigned adult mice to five conditions for ~40 days: (1) water-maze learning, (2) yoked swimming (forced exercise controls), (3) voluntary running wheel, (4) enriched environment, and (5) standard housing. Water maze training and forced swimming had no effect on cell proliferation or neurogenesis. Voluntary running doubled surviving newborn cells, similar to the effect of the enriched environment. However, only runners showed increased proliferation at day 1 (6,773 cells *vs.* ~ 4,000 in other groups), whereas enrichment increased only survival (42%–46% survival in most groups *vs.* 85% in enriched, 56% in runners). The study suggests that voluntary physical exercise alone can be sufficient to enhance hippocampal neurogenesis. Still, the numbers suggest that enrichment’s superior cell survival rate does not dismiss its unique potential.

The implications of exploring whether mazes can support adult hippocampal neurogenesis are not only useful for laboratory studies on animal models but also for humans since adult hippocampal neurogenesis continues throughout the lifespan in mammals including both humans and rodents (Alonso et al., 2024; Elliott et al., 2025; Zhang et al., 2023). The discovery that new neurons are generated in the adult hippocampus in rodents (Altman and Das, 1965; Kaplan and Hinds, 1977), and later in humans (Eriksson et al., 1998; Moreno-Jiménez et al., 2021; Spalding et al., 2013), necessitates bridging the gap in environmental enrichment research between both species. Adult hippocampal neurogenesis can be therapeutic for cognitive decline, Alzheimer’s disease, depression, and repeated stress (Chen et al., 2025; Surget and Belzung, 2022), which further strengthens the need to bridge this gap since adult hippocampal neurogenesis persists in humans into the tenth decade of life (Dumitru et al., 2025; Moreno-Jiménez et al., 2021; Tobin et al., 2019). There are clues that the human brain’s hippocampus, and potentially adult hippocampal neurogenesis, is stimulated by spatial complexity. Navigating complex landscapes and cities is associated with protected hippocampal volume and lower risks of Alzheimer’s disease (Lövdén et al., 2012; Shin et al., 2024), and it is already established that adult hippocampal neurogenesis in humans drops in patients with Alzheimer’s disease (Moreno-Jiménez et al., 2019; Tobin et al., 2019). To address this gap, the architectural spatial complexity index (A-SCI) tool, which was developed and theoretically validated in a previous study on human buildings (Khalil, 2026), provides a quantitative metric for assessing the pro-neurogenic potential of environments based on layout complexity and diversity and count of points of interest (POIs). The A-SCI can assess human and rodent layouts solely based on layout complexity independently from the effect of visual cues, which provides a feasible comparative assessment of spatial complexity at this stage for theoretical, laboratory, or built mazes for humans in gardens or museums. The current study extends the application of the approach and does not represent a direct replication.

## 2 Methods

### 2.1 The architectural spatial complexity index (A-SCI)

This study uses the architectural spatial complexity index (A-SCI), which was introduced earlier (Khalil, 2026). The A-SCI integrates spatial features organized into equally-weighted categories: layout complexity variables (*n* = 7) and points of interest variables (*n* = 7). Yet, the A-SCI can still provide assessment for each category independently. Layout complexity includes average node degree, intersection count, total segment length, number of segments, average segment length, average circuitry, and self-loop proportions. POIs represent the count of distinct subcategories, which are easier to understand in rodent housing such as tunnels, houses/domes/igloos, swings, crawl structures, stairs/ladders/slopes, running wheels, and toys. Items in each category are quantified respective to the definition of the item, and the values are used to generate the relative index scores based on the evenness and abundance of values in each data set. The A-SCI is calculated using a standardized formula that calculates the scores based on the square root of the normalized Entropy multiplied by the maximum ratio, an equation developed by Shin et al. (2024). The A-SCI, however, is modified to provide scores ranging from 0–1, where higher A-SCI scores indicate greater spatial complexity.

### 2.2 Dataset

The dataset from the previous validation study included mazes (six variants of the Marlau cage and the Hamlet complex maze), standard housing examples from multiple studies, and POI-based environmental enrichment examples (Khalil, 2026). Sample size was determined based on the availability of description and illustrations of the maze or enrichment in order to be able to rasterize the image in AutoCAD and to measure or count the values. In the present study, this dataset is substantially expanded to include eight additional maze types selected from a recent comprehensive review by Wijnen et al. (2024): T-maze: A simple three-arm maze with one stem and two choice arms (Krausz et al., 2023); Repeated T-maze: Multiple T-maze units connected in series (Tolman and Nyswander, 1927); Repeated Y-maze: Multiple Y-maze units connected in series (Warden, 1929); Hampton Court maze: A complex hedge maze configuration scaled for rodents (Small, 1901); Radial 8-arm maze: Eight arms radiating from a central platform (Dale and Inis, 1986); Crossword maze: A grid-based maze with intersecting pathways (McNamara et al., 2014); Lattice maze: A regular grid pattern with multiple choice points (Okaichi, 1996); Hex maze: A hexagonal grid-based maze with six-way junctions (Alonso et al., 2021). The dimensions of each maze were obtained from their respective studies.

Dimensional specifications for each maze were obtained from the respective published studies. Layouts were rasterized in Autodesk AutoCAD 2026 to identify the values for each of the seven layout complexity variables required for A-SCI calculation.

### 2.3 Comparative analysis framework

To control for the independent effect of layout complexity of mazes, all mazes were first analyzed with POI variables set to zero. Consequently, three comparative assessments were conducted to:

1. Test the difference in the relative A-SCI scores between environmental enrichment (with the Marlau cage, Hamlet maze, and POI-based enrichment combined) and standard housing.
2. Test the difference in the relative A-SCI scores between mazes demonstrated to be pro-neurogenic (Marlau cage and Hamlet maze), POI-based enrichment, and standard housing.
3. Assessment of the difference of the relevant A-SCI scores between the maze layouts only. This analysis allows relatively generating spatial complexity scores for mazes solely based on their layouts. Based on the identified difference in A-SCI scores between the studied mazes, it was not feasible to group them and conduct assessment between mazes.

### 2.4 Statistical analysis

Relative A-SCI scores were calculated for each step of analysis using the equations previously published (Khalil, 2026). Descriptive statistics, including means and standard deviations, were computed for each group. Normality of distributions was assessed using the Shapiro–Wilk test. One-way Analysis of variance (ANOVA) with Games-Howell *post hoc* test was conducted to test for significant differences between groups if normality was not violated; otherwise a nonparametric, the independent-samples Kruskal-Wallis test, was used with pairwise comparisons. A non-parametric test is used when violations for normality and homogeneity of variances were detected. Statistical significance was set at *p* < 0.05. All analyses were performed, using SPSS 30.

## 3 Results

**Figure 1:**
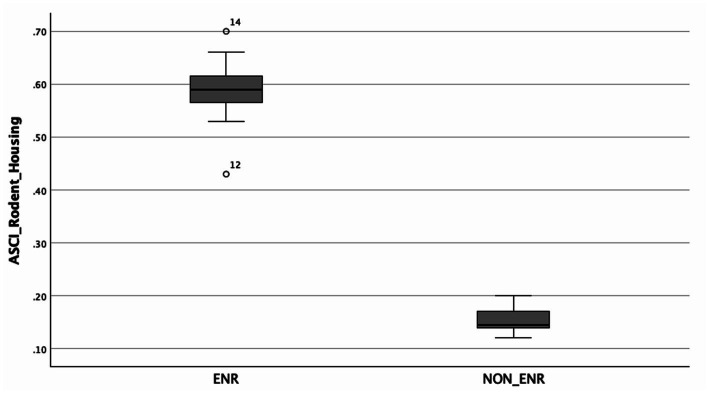
Boxplot of A-SCI scores across the two groups: ENR (*n* = 11) and NON-ENR (*n* = 6). Circles denote mild outliers.

**Figure 2:**
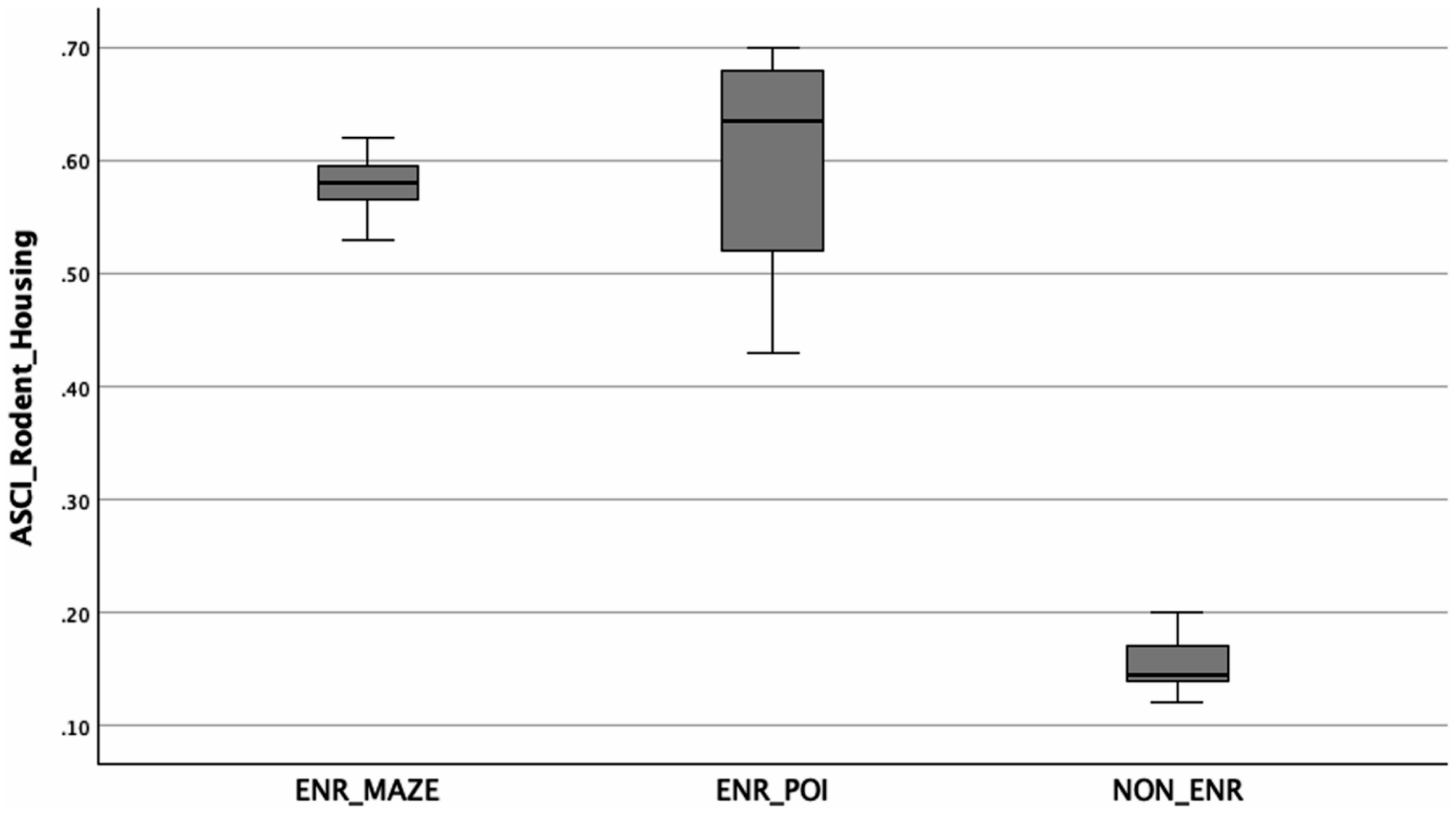
Boxplot of A-SCI scores across the three groups: ENR_Maze (*n* = 7), ENR_POI (*n* = 4), and NON-ENR (*n* = 6).

First, based on the obtained theoretical values (Table 1), the A-SCI scores were calculated. An independent *t*-test was used to compare mean A-SCI scores between groups, revealing a significant difference between the combined enrichment group and the standard housing group [*t*(15) = 14.38, *p* < 0.001, one-and two-tailed], with the combined enrichment (ENR) group (*M* = 0.5864, *SD* = 0.0699) showing higher A-SCI scores than the standard housing (NON-ENR) group [*M* = 0.1533, *SD* = 0.02805; mean difference = 0.43303, 95% CI (0.36886, 0.49720)]. Effect sizes indicated a very large difference [Cohen’s *d* = 7.30, 95% CI (4.514, 10.053)]. This outcome confirms that the ENR group scores significantly higher on the A-SCI than the NON-ENR group, supporting that the A-SCI tool is able to differentiate between pro-neurogenic housing and standard housing (Figure 1). Subsequently, the one-way Welch’s ANOVA test revealed a statistically significant difference in scores among the three groups (ENR_Maze, ENR_POI, and NON_ENR), *F*(2, 6.387) = 330.996, *p* < 0.001. The Games-Howell *post hoc* analysis (Table 2), however, showed that there is no significant difference in the A-SCI scores between enrichment using POIs or a maze, suggesting that their overall complexity is not significantly different Figure 2.

**Table 1:** The extracted raw values from non-enriched and pro-neurogenic enriched rodent housing layouts.

EE*	Layouts	Layout variables in rodent housing***							Points of interest variables in rodent housing****						
		1	2	3**	4	5**	6	7	8	9	10	11	12	13	14
1	Marlau_AA (Fares et al., 2013)	2.14	8	910	14	65.0	1.69	0.50	0	0	0	0	2	0	0
1	Marlau_BB (Fares et al., 2013)	2.20	9	910	13	70.0	1.56	1.08	0	0	0	0	2	0	0
1	Marlau_CC (Fares et al., 2013)	2.29	4	910	8	113.8	1.80	0.88	0	0	0	0	2	0	0
1	Marlau_DD (Fares et al., 2013)	2.36	7	910	11	82.7	1.61	0.82	0	0	0	0	2	0	0
1	Marlau_EE (Fares et al., 2013)	2.18	6	910	14	65.0	1.68	0.50	0	0	0	0	2	0	0
1	Marlau_FF (Fares et al., 2013)	2.27	9	910	13	70.0	1.66	0.77	0	0	0	0	2	0	0
1	Hamlet (Crouzier et al., 2018)	2.27	6	760	15	50.7	1.05	0.40	0	0	0	0	0	1	0
0	Red_Tunnel (Oatess et al., 2021)	1.00	0	112	1	112.0	1.00	0.00	0	0	0	0	0	0	0
0	Oatess et al. (2021)	1.00	0	112	1	112.0	1.00	0.00	1	0	0	0	0	0	0
0	Funabashi et al. (2023)	1.00	0	56	1	56.0	1.00	0.00	0	0	0	0	0	0	0
2	Funabashi et al. (2023)	1.00	0	180	1	180.0	1.00	0.00	3	3	1	1	1	0	0
2	Ramírez-Rodríguez et al. (2022)	1.00	0	78	1	78.0	1.00	0.00	6	0	0	0	0	1	3
0	Ramírez-Rodríguez et al. (2022)	1.00	0	78	1	78.0	1.00	0.00	0	0	0	0	0	0	0
2	Wang et al. (2020)	1.00	0	100	1	100.0	1.00	0.00	1	1	1	1	1	1	4
0	Wang et al. (2020)	1.00	0	54	1	54.0	1.00	0.00	0	0	0	0	0	0	0
2	Sakhaie et al. (2020)	1.00	0	150	1	150.0	1.00	0.00	2	1	1	0	2	1	2
0	Sakhaie et al. (2020)	1.00	0	105	1	105.0	0.00	0.00	0	0	0	0	0	0	0

*0 = standard housing (non-enrichment); 1 = Enrichment through layout-based complexity (maze); 2 = enrichment through points of interest complexity (POI). **length measurements are presented in centimeters. A-SCI was calculated twice, when length is measured in centimeters and meters, resulting in the same scores. *** Layout variables: 1 = Average node degree; 2 = Intersection count; 3 = Total segment length; 4 = Number of segments; 5 = Average segment length; 6 = Average circuitry; 7 = Self-loop proportions. ****POIs: 8 = tunnels; 9 = houses, domes, igloos; 10 = swings; 11 = crawl; 12 = stair, ladder, slope; 13 = wheel; 14 = toys.

**Table 2:** Games-Howell *post-hoc* multiple comparisons.

(I)	(J)	Mean difference (I-J)	Std. error	Sig.	95% Confidence interval	
					Lower bound	Upper bound
ENR_Maze	ENR_POI	−0.02143	0.06059	0.935	−0.2642	0.2214
	NON-ENR	0.42524*	0.01588	<0.001	0.3822	0.4682
ENR_POI	ENR_Maze	0.02143	0.06059	0.935	−0.2214	0.2642
	NON-ENR	0.44667*	0.06067	0.008	0.2043	0.6891
NON-ENR	ENR_Maze	−0.42524*	0.01588	<0.001	−0.4682	−0.3822
	ENR_POI	−0.44667*	0.06067	0.008	−0.6891	−0.2043

*The mean difference is significant at the 0.05 level.

Afterwards, this study aimed to explore the relative A-SCI scores of the Hamlet Complex Maze and the Marlau cage compared to other mazes to understand whether they score differently and to inform future research if other mazes score higher. Table 3 presents the values obtained for each maze.

**Table 3:** Values for layout complexity variables in each maze.

Mazes	Layout variables in rodent housing*							Points of interest variables in rodent housing*						
	1	2	3	4	5	6	7	8	9	10	11	12	13	14
Marlau_AA	2.14	8	910	14	65.0	1.69	0.50	(all POIs purposely set to 0 to control for the independent effect of the maze architecture-based layout complexity variables, while maintaining the ratio between layout complexity and POIs required to calculate the A-SCI)						
Marlau_BB	2.20	9	910	13	70.0	1.56	1.08							
Marlau_CC	2.29	4	910	8	113.8	1.80	0.88							
Marlau_DD	2.36	7	910	11	82.7	1.61	0.82							
Marlau_EE	2.18	6	910	14	65.0	1.68	0.50							
Marlau_FF	2.27	9	910	13	70.0	1.66	0.77							
Hamlet	2.27	6	760	15	50.7	1.05	0.40							
Lattice	1.94	20	1,339	52	25.8	1	0.02							
Hex	3.00	12	600	18	33.33	1	2.78							
Crossword	2.06	13	707	34	20.80	1.02	0							
Radial 8-arm	1.18	1	639	8	117	1	0							
Hampton court	2.07	7	3,095	16	186.44	2.37	0.06							
Repeated Y (4)	1.80	4	198	9	22	1	0							
Repeated Y (10)	1.91	10	462	21	22	1	0							
Repeated T (6)	1.86	6	513	13	39	1	0							
T maze (1)	1.50	1	117	3	39	1	0							

***Layout variables: 1 = Average node degree; 2 = Intersection count; 3 = Total segment length; 4 = Number of segments; 5 = Average segment length; 6 = Average circuitry; 7 = Self-loop proportions.

**Figure 3:**
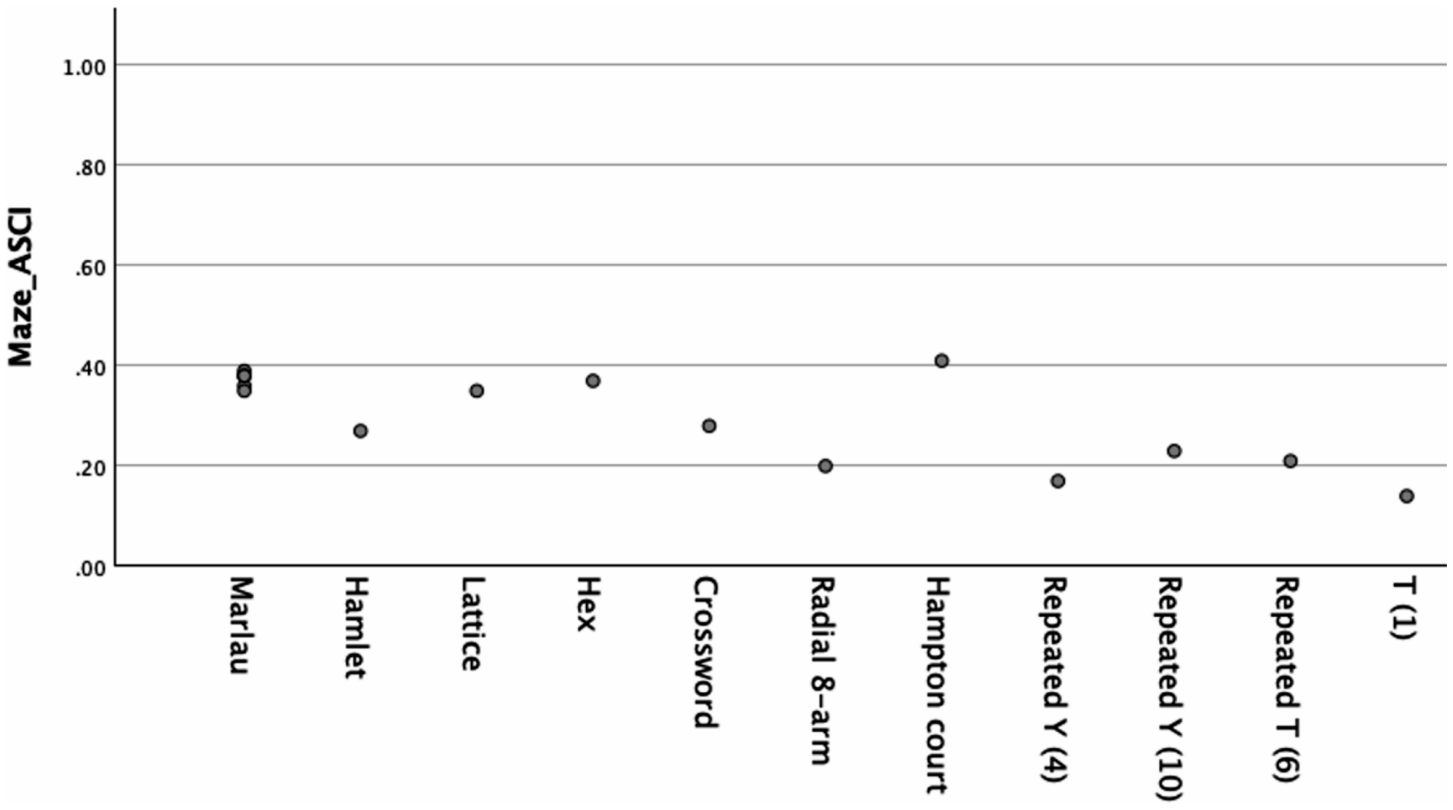
Relative A-SCI scores for maze architectures.

The relative A-SCI scores for mazes were analyzed independently (Figure 3). This analysis included 16 maze configurations: six variants of the Marlau cage (AA through FF) in the same category, the Hamlet maze, the Lattice maze, the Hex maze, the Crossword maze, the Radial 8-arm maze, the Hampton Court maze, two variants of the Repeated Y-maze (4 and 10 repetitions), the Repeated T-maze (6 repetitions), and the single T-maze. The graph shows variation in complexity across maze types.

Interestingly, the Hampton Court maze achieved the highest A-SCI score (0.41), the Hex maze achieved the second-highest score (0.37), the interchangeable six configurations in the Marlau cage, which is suggested to support adult hippocampal neurogenesis, clustered together with scores ranging from 0.35 to 0.39, demonstrating relatively consistent architectural complexity across their different variants. The Lattice maze architecture scored similar to the lowest threshold of the Marlau cage maze configurations (0.35), while Crossword maze scored 0.28, slightly higher than the pro-neurogenic Hamlet complex maze architecture that scored 0.27. The simpler maze designs showed notably lower scores. The Radial 8-arm maze architecture scored 0.20, while the repeated 10-Y-mazes, 4-Y-mazes and 6-T-mazes scored 0.23, 0.17, and 0.21, respectively, and the single T-maze scored 0.14.

These results demonstrate that both the Marlau cage and Hamlet maze, which reported their effectiveness to support adult hippocampal neurogenesis despite the confounding effect of running wheels, demonstrate equal complexity similar to POI-based enrichment, and relatively higher to other forms of mazes except the aforementioned examples.

## 4 Discussion

This study demonstrates that the A-SCI tool can significantly differentiate between environmental enrichment conditions and standard housing conditions (*p* < 0.001, Cohen’s *d* = 7.30), and post-hoc results hypothesize no difference between enrichment through mazes or enrichment through POIs. This finding holds theoretical validity knowing that previous research on rodents suggests the same negligible difference (Rosenzweig et al., 1978). This finding suggests but urges future research to validate that the Marlau cage and Hamlet complex maze both enhanced adult hippocampal neurogenesis independently from the effect of running wheels (Crouzier et al., 2018; Fares et al., 2013). Furthermore, this article suggests that the A-SCI tool transcends research on environmental enrichment for adult hippocampal neurogenesis from laboratory to human environments, reducing the gap by facilitating translational research. Hampton Court Maze emerges as a promising comparative example not only for rodents but also for humans.

The Hampton Court maze, a rodent-scale adaptation of an actual human maze built in 1690 (Bounford, 2022), achieved the highest A-SCI score (0.41) among all maze architectures examined, including the Marlau cage and Hamlet maze that suggested to support adult hippocampal neurogenesis in rodents combined with the effect of running wheels (Crouzier et al., 2018; Fares et al., 2013). This finding has profound implications for translational research. If the most complex rodent maze is one modelled on human context, the Hampton Court Maze, which is not even affected by way finding or navigational cues that may dilute complexity, future research can use the Hampton Court Maze as a common example shared between humans and rodents to compare and contrast adult hippocampal neurogenesis. Translational research is needed, and it can be facilitated by the A-SCI tool, knowing that apartment living is significantly lower in complexity than multi-story houses and Hampton Court Maze in humans (Khalil, 2026).

Future research should pursue several directions. First, validate the neurogenic potential of the Hamlet Complex Maze and Marlau Cage independently from the effect of running wheels. Second, compare whether Hampton Court Maze for rodents, Hamlet Complex Maze, and Marlau Cage have different effects on adult hippocampal neurogenesis in rodents based on the different scores of complexity generated by the A-SCI tool. Third, comparative studies across rodent species (rats versus mice) and strains would clarify whether A-SCI rankings remain consistent or require species-specific calibration. Fourth, longitudinal studies using the A-SCI tool can track where humans spend their time and can help observe whether variability of adult hippocampal neurogenesis markers between subjects in postmortem samples can be explained through the spatial complexity scores generated for buildings via the A-SCI tool. This is supported by previous research showing that navigating complex landscapes protects hippocampal volume of humans (Lövdén et al., 2012), geospatial complexity is associated with hippocampal volume and lower risk of Alzheimer’s disease (Shin et al., 2024), and increased neighborhood walkability is associated with similar outcomes independently from the effect of physical activity (Cerin et al., 2018).

However, several limitations of this study warrant acknowledgment. The current study based its assessment on two-dimensional measurements, and it would be useful in future research to explore how three-dimensionality using levels, stairs or ramps might influence the A-SCI scoring and the outcome of the enrichment in rodent models. Currently, there is no evidence to explain the mechanisms through which mazes can stimulate neurogenesis in rodents; however, there are two factors that should be taken into account in forthcoming comparative studies between rodents and humans. Spatial experiences in complex environments can be affected by several factors such as cognitive loads and visuospatial demands. First, a maze configuration that appears complex from a human perspective may not impose equivalent cognitive load on a rodent or vice versa. Additionally, familiarity plays a key role in determining cognitive load. Where studies on rodents typically require changing the maze configuration, human buildings, landscapes and cities have fixed networks that do not change. Yet, rodents do not change their housing environments unlike humans who navigate different cities, landscapes and buildings, an opportunity that can itself become the needed change. Second, the visuospatial demands of navigating complex environments may be substantially reduced by the availability of environmental cues, which has important implications for comparing maze effects across species. In human built environments, at both the urban and interior levels, spatial navigation is facilitated by signage, colors, landmarks, and street names that provide explicit way finding information. In contrast, rodent maze paradigms typically present impoverished visual environments with limited salient cues, requiring animals to rely more heavily on path integration and self-generated spatial representations. This fundamental difference suggests that cognitive demands may be inversely related to the richness of available cues.

The goal of this study is bidirectional through its translation ability using the A-SCI tool that differentiates environmental enrichment from non-enrichment conditions. The A-SCI tool does not predict causal claims but rather provides a useful measurement to explore the sophisticated relationship between complex layouts for rodents, humans, and between both species. This study shows that rodent studies can be representative of human buildings, landscapes and layouts if future research explores the pro-neurogenic potential of mazes that can be quantified using the A-SCI tool. This approach encourages translational research and advances research on adult hippocampal neurogenesis in both humans and rodents.

Future rodent studies should develop microcosms that more accurately replicate the structural complexity of human-built environments, including scaled models of buildings, landscapes, and urban layouts, to better understand how architectural and spatial complexity influences adult hippocampal neurogenesis. Current rodent maze paradigms bear limited resemblance to human environments, and they remain limited by the confounding effect of running wheels. Such ecologically valid rodent microcosms would allow experimental isolation of layout complexity from physical exercise by comparing neurogenesis across environments of equivalent size but varying topographical intricacy. Using the A-SCI tool, this approach would not only enhance the translational relevance of rodent neurogenesis studies to humans, but also provide mechanistic insights into whether the spatial cognitive demands of navigating complex built environments contribute meaningfully to hippocampal neurogenesis beyond the well-established effects of physical activity alone.

## 5 Conclusion

This study quantified spatial complexity across rodent maze architectures using the Architectural Spatial Complexity Index, revealing substantial variation in environmental enrichment potential between maze types. The A-SCI tool successfully differentiated enriched from non-enriched housing and demonstrated theoretical equivalence between maze-based and object-based environmental enrichment, validating earlier research on rodents finding almost similar effect on cortical thickness. Hampton Court maze, a rodent-scale adaptation of an actual human maze, achieved the highest complexity score, surpassing both the Marlau cage and Hamlet maze that have demonstrated pro-neurogenic effects in previous studies. This finding has profound implications for translational research: the most complex rodent maze examined is one modelled on human architecture, providing a possible comparative paradigm for studying adult hippocampal neurogenesis across species. Future research should validate whether maze architecture complexity independently predicts neurogenic outcomes when controlling for confounding factors such as running wheels, compare neurogenesis across mazes with different A-SCI scores, and develop ecologically valid rodent microcosms that better represent human built environments. By providing a neuroscience-informed, quantitative framework for assessing environmental complexity, this study advances translational research on adult hippocampal neurogenesis and its implications for cognitive health in both humans and rodents.

## Data Availability

The original contributions presented in the study are included in the article/supplementary material, further inquiries can be directed to the corresponding author.
